# Exploring senior managers’ perceptions of the COVID-19 Crisis in Iran: a qualitative content analysis study

**DOI:** 10.1186/s12913-021-07108-6

**Published:** 2021-10-09

**Authors:** Mostafa Bijani, Shahnaz Karimi, Aliasghar Khaleghi, Yousef Gholampoor, Zhila Fereidouni

**Affiliations:** 1 Department of Medical Surgical Nursing, Fasa University of Medical Sciences, Fasa, Iran; 2 Department of Medical Education, Medical Education Research Center, Fasa University of Medical Sciences, Fasa, Iran; 3 NonCommunicable Diseases Research Center (NCDRC), Fasa University of Medical Sciences, Fasa, Iran

**Keywords:** Health Services Research, COVID- 19, Crisis management. Qualitative research

## Abstract

**Background:**

Identification of the experience of senior managers in tackling biological crises can be a roadmap for future crisis management planning. The aim of the present study was to investigate the experiences of senior managers during the COVID-19 crisis.

**Methods:**

This is a descriptive qualitative research. Data were collected using in-depth and semi-structured individual interviews. Accordingly, 20 senior managers of medical universities with experience in managing the COVID-19 crisis were enrolled in the study using purposive sampling. Data were collected from February 2020 to May 2021. For data analysis, qualitative content analytical approach was used.

**Results:**

According to the results, 4 main themes and 10 sub-themes were obtained; they included dealing with issues and challenges in the face of COVID-19 disease (Structural challenges, Cultural challenges, Educational challenges, COVID-19 complexity); individual and managerial competencies (Individual competencies, Managerial competencies); comprehensive, accountable, and efficient management (Comprehensive and accountable management, efficient management); and professional and organizational self-efficacy (Professional self-efficacy, organizational self-efficacy) were obtained.

**Conclusions:**

In the present study, a number of senior managers’ experiences in the COVID-19 crisis management were identified. Managers and policymakers of the health system are suggested to use the results of the present study to effectively manage the crisis and improve crisis management in various health-related areas by providing an effective cultural and organizational context.

## Introduction

Crisis management is a major part of strategic management and ensures the success of an organization; if it is neglected, it leads to irreparable damage to the country [1]. A review of the literature showed that crises for the academic community were limited to natural disasters such as floods and earthquakes, and, unfortunately, there is limited research on “crisis management”. However, there are fewer studies on crises, such as the outbreak of an emerging viral infectious disease, including the COVID-19 [2]. The emerging COVID-19 disease started in China in 2019; it soon became a pandemic and led to the death of millions of people worldwide [3]. Although COVID-19 initially threatened the health sector, it quickly spread to other areas. It can be said that the crisis is the first borderless crisis in all biological, social, economic, and political dimensions in the world [4]. Despite the numerous problems caused by the COVID-19 crisis, it is important to evaluate health crisis management since it can provide rich experiences for other crises. Since there are different management levels, conditions underlying the crisis, types of crisis, and severity of the pandemic, it seems necessary to study the experiences in each crisis separately, especially the COVID-19 [5]. Qualitative studies are important for searching, describing, and gaining in-depth insight [6].

## Background in Iran

The attack of COVID-19 in Iran was confirmed on 18 February 2020 and imposed suppression and lockdown in all provinces. The government of Iran established specialized health centers to control COVID-19. Statistics show that the highest number of illnesses and deaths has occurred in Iran in Aug. 23, 2021, with 4,677,114 cases of COVID-19 and 102,038 deaths. The financial constraints of the government of Iran to protect the nation financially during the lock down have rendered the control of COVID 19 complicated and impossible [7].

Many Iranian researchers have, thus, focused on the issue of Coronavirus. A qualitative study by SoleimanvandiAzar et al. (2021) revealed various individual, socio-cultural, economic, and structural factors which influenced the non-observance of health advice and a low number of preventive behaviors among people with high-risk jobs [8]. A study on clerics in confrontation with the COVID-19 Crisis in Iran demonstrates that clerics can be effectively used during health crises. However, they can act on the opposite front and contribute to disease transmission, or disrupt the scientific interventions performed for disease prevention and treatment [9].

Corona crisis is influenced by various cultural, economic and social factors, and there are many problems in managing this crisis. Additionally, an understanding of senior managers’ experiences on COVID-19 management is important because it will help the authorities to identify the factors that improve the quality of management. One of the first countries whose population were infected with the virus after China was Iran; however, the consequences of the pandemic for Iranian care providers, especially senior managers, have not been studied so far. These challenges and their coping strategies are greatly dependent on the experiences of senior managers with pandemic crisis. It is necessary to examine this process with a holistic and qualitative approach. Therefore, the aim of the present study was to explore the experiences of senior managers of medical universities on crisis management in Iran.

## Methods

The present study used a descriptive qualitative design which is an effective method to obtain insight into a research question and establish the subjects’ perception of who, what, place of events, or experiences treatment [10]. When little is known about the informants’ perception of a phenomenon, a descriptive qualitative design can help the researchers use human experiences in their unique context [11]. The reporting of the study was based on the consolidated criteria for reporting qualitative research (COREQ) checklist [12].

### Participants

In this research, 20 senior managers were first selected as the key informants based on purposive sampling, and three university chancellors were interviewed based on theoretical sampling until data saturation was achieved. The selection criteria included an experience of at least 6 months in managing the COVID-19 crisis as a senior manager and willingness to participate in the research and express their personal experiences. Twenty individual and semi-structured interviews were used to collect the data.

### Data collection

Purposive sampling was performed, and data were collected from February 2020 to May 2021. To collect the data, we used semi-structured face-to-face interviews. The location and time of the interviews were determined as agreed by the participants. The interviews were decided to be conducted at Fasa University of Medical Sciences. The interviews were performed by one of the researchers who was experienced in qualitative research (SHK). Further supports were also provided by other team members who were experienced in management (ZF). Interviews began using general questions, including “Describe your managerial experience with the COVID-19 crisis management”; then, we continued with more specific questions, such as “What are some challenges and barriers to the COVID-19 crisis management based on your experience?“ and “What solutions do you suggest to improve the COVID-19 crisis management?“ In order to increase the clarity of information, probing questions such as “Can you explain more?“, “What do you mean?“, and “Can you give an example?“ were raised. The interview process was followed according to the main objective of the research. The interviews lasted between 35 and 60 min. Sampling was purposeful, and theoretical saturation was achieved by conducting 20 interviews and the main themes and sub-categories were formed. The theoretical saturation is considered as the gold standard at the end of sampling in qualitative research. This situation occurs when no new code can be added to the data; then, saturation is attained, and the researchers can stop the interview process [13]. After the 16th interview, there were no new codes and categories generated from the interviews. Therefore, it was deemed that the data collection had reached a saturation point. We continued data collection for four more interviews to ensure and confirm that there were no new categories emerging.

### Data analysis

Data analysis was performed at the same time as data collection immediately after each interview. Data analysis was carried out using Graneheim’s and Lundman’s conventional qualitative content analysis [14]. In the first step, the research team first listened to the interviews several times and then transcribed them on paper at the end of each interview. In the second step, the interview scripts were read for several times to gain a general understanding of the participants’ statements in line with the study objectives. In the third step, all the texts of the interviews were read word by word with great care and patience; then, the initial codes of the meaning units were extracted by the research team. In the fourth step, the researchers merged and categorized the codes, which were similar in meaning and concept and could be placed into a subcategory due to their association with each other. In the fifth step, subcategories were placed in the main categories, which were conceptually more comprehensive and abstract. Finally, in a joint session, the whole data analysis process was shared, and the opinions of all the authors of the article were used analysis [14]. The collected data were finally analyzed in MAXQDA v. 2007.

### Rigor

To enhance the accuracy of the study results, we used Lincoln’s and Guba’s criteria [15]. Accordingly, to ensure credibility, the researchers applied prolonged engagement with data, member checking, and peer debriefing. For member-checking, 3 nurses were presented with a copy of the encoded interviews; they confirmed it. To perform peer-checking, the researchers asked five experts to analyze and observe the process of data analysis and validate the codes and categories. The dependability of the study results was enhanced through describing the methods used to code the concepts and themes and presenting textual and audio- data. Also, two members of the research team examined the findings individually and then discussed them to resolve any possible disagreements to ensure dependability. To ensure that the results were confirmable, the researchers showed the encoded data to the participants to verify the accuracy of the extracted categories and subcategories. The conditions of the respondents and the way they were interviewed were clearly described to add to the transferability of the study results. The researchers also tried to select the study subjects solely based on the objectives of the study and free of bias. Data were analyzed as soon as they were collected to help the researcher be aware of the principles of the research. Transferability was ensured by providing a comprehensive description of the subject of the study, the participants’ characteristics, and methods of data collection and data analysis, along with documented examples of the participants’ quotes.

### Ethical considerations

 All participants gave written informed consent to participate in the study. The present study was conducted in accordance with the principles of the revised Declaration of Helsinki, a statement of ethical principles which directs physicians and other participants in medical research involving human subjects. The study objectives were first explained to the participants and written informed consent was obtained from those who wished to participate in the study. Permission was also obtained to record the interview with an audio recorder. The participants were reassured about the principles of confidentiality and anonymity, the voluntary entry into the study, and the freedom to withdraw from the study at any time without any consequences. The time and place of the interview were chosen in coordination with the participants. The study was approved by the local Ethics Committee of Fasa University of Medical Sciences, Fasa, Iran (Ethical code: IR.FUMS.REC.1399.012).

## Results

A total of 20 senior managers participated in the present study. Among them, there were 14 (70 %) male and 6 (30 %) female. The mean ± SD of the participants’ age was 47.38 ± 10.4 years. Participants included staff and clinical managers with a work experience of 2 to 15 years in crisis management. Participants in the crisis management course had positions such as the chancellor of the university, vice-chancellor for treatment, vice -chancellor for food and drug department, health vice chancellor, education vice-chancellor, vice chancellor for student cultural affairs, directors of the hospitals, and healthcare service managers. All demographic characteristics of the participants are presented in Table 1.

**Table 1:** The demographic characteristics of senior managers who participated in the study

Participants	Gender	Age	Management level	work experience	Management experience
1	Male	51	chancellor of the university	25	15
2	Male	49	vice chancellor for treatment	20	9
3	Female	42	vice chancellor for food and drug department	12	11
4	Male	41	vice chancellor of health	12	5
5	Male	55	vice-chancellor for education	14	13
6	Male	51	vice chancellor for student cultural affairs	22	15
7	Male	39	directors of the hospital	9	7
8	Female	37	clinical supervisor	17	3
9	Female	38	nursing service manager	19	5
10	Male	40	healthcare service managers	17	8
11	Male	41	vice-chancellor for Finance and Support	27	5
12	Male	44	director of Medical Emergency and Accident Management Center	13	10
13	Male	39	manager of the Crisis Committee	19	13
14	Male	44	hospital manager	18	12
15	Female	47	infectious disease specialist	16	5
16	Female	46	epidemiologist	11	4
17	Male	35	health manager in disasters and emergencies	7	2
18	Male	41	Vice-chancellor for Research Affairs	15	5
19	Male	42	governor	9	2
20	Female	39	social medicine specialist	14	7

**Table 2:** Themes, main sub-themes, and basic concept codes extracted

Themes	Sub-themes	Basic concept codes
Dealing with issues and challenges in the face of COVID-19 disease	Structural challenges	Lack of medical equipment, Lack of manpower, Lack of physical space for hospitalization
	Cultural challenges	Lack of belief in the disease and health protocols, Absence of an authoritative voice, The difficulty in cooperation of city officials and people. Negligence of health protocols
	Educational challenges	Ineffectiveness of crisis management training courses, Lack of knowledge logistics about COVID-19
	COVID-19 complexity	Confusion due to unknown disease, Destructive flood. Little monster,The real battlefield, Annoying tsunami in all aspects of socio-economic health
Individual managerial competencies	Individual competencies	Far-sightedness, Perseverance and seriousness, Self-sacrifice,Experience and skills, Responsibility, Compassionate work
	Managerial competencies	Formation of joint teams. Use of collective wisdom, Optimal use of time, Communication management, Predict and act fast
Comprehensive, accountable and Efficient management	Comprehensive and accountable management	Timely notification and awareness, Utilization of the capabilities of key people in the society, Full-time response, Accompanying people, Use of all available capacities in the society, Mobilization of faculty members and field activities
	Efficient management	Physical space management in the hospital, Equipment management, Manpower management, Timely management with knowledge and updatedness,
Professional and organizational self-efficacy	Professional self-efficacy	Skills promotion, Feeling empowered
	Organizational self-efficacy	A reduction in need and dependency. Forming scientific groups. Strengthening the intermediate managers

Participants’ experiences in the COVID-19 crisis management led to the extraction of four main themes, including individual and managerial competencies; comprehensive, accountable, and efficient management; and professional and organizational self-efficacy in the crisis management. Table 2 shows the themes, subthemes, and extraction codes.

### Dealing with issues and challenges in the face of the COVID-19crisis

The first main category of dealing with issues and challenges in the face of the COVID-19 crisis included four subcategories: structural challenges, cultural challenges, educational challenges, and the COVID-19 complexity.

#### 1.1 Structural challenges

Summary of the participants’ experiences in this subcategory included lack of resources and equipment including lack of ventilators, protective equipment such as masks, gloves and a disinfectant, medications, manpower, and physical space in the hospital. The unavailability of the required equipment caused numerous self-care and self-protection problems for the treatment team.

*“There was no sufficient personal protective equipment for the staff, companions, and public. There were no sufficient hospital ventilators for the patients. The hospital ventilation system was integrated. We needed to separate the ventilation system to prevent other patients from getting infected. If we had reached the peak of the disease, there was no sufficient space to accommodate the patients.“ (Participant 3).*

#### 1.2 Cultural challenges

According to the participants’ experience, one of the cultural challenges in the COVID-19 crisis management was delayed acceptance of the disease by managers and officials, which in turn led to wasting much time to inform and convince them. This made it difficult to coordinate the efforts to manage the crisis. People also behaved differently. Some did not believe in the disease and did not observe health protocols. Fear and panic had disrupted their daily living activities. Some people considered it a taboo to be infected with the disease due to the lack of understanding of the disease and tried to hide their infection. Another cultural challenge related to the COVID-19 management was the absence of an authoritative voice. In other words, there were different conversations about the treatment, transmission route, and vaccination of different people at the senior executive level in the world.

*“City officials did not understand the disease and did not prioritize it over other affairs. Some people do not believe in it until they themselves get disease and die. “There is still no coherence. When there is not an authoritative voice, it causes anxiety, distress, and lack of public trust” (Participant 1).*

#### 1.3 Educational challenges

Based on the participants’ experience, crisis management training courses could not help them in the current situation. On the other hand, at the beginning of exposure to the disease at the bedside, most managers sought to adopt individual procedures in coronary control, and the principles of teamwork were not considered. Also, the principles of medical care were not taken into account while delivering health services, which indicated a weakness in education.

*“If the principles of treatment are not observed, it will be like a loose structure that collapses with a wind. The fact that the staff of the intensive care units do not know the principles of patient care is the result of disregarding the necessary training. Passive defense training sessions did not provide the necessary preparation. “Medical work is not an individual job. A COVID-19 inpatient with a respiratory problem needs to be visited by a specialist. The structure was wrong, and the patient’s round was carried out by a specialist.“ (Participant 5).*

Another participant stated that:

*“There is a lack of knowledge logistics about the COVID-19, and there were no answers to many questions that can be answered in a timely manner through research to be effective for patient care. More than a year has passed since the onset of the COVID-19; we have experienced several peaks and many questions still remain unanswered. At this center, almost all patients under the age of 50 who died of the COVID-19 were obese, because every obese person died after COVID-19 infection” (Participant 18).*

#### 1.4 COVID-19 complexity

Most of the participants stated that the pandemic created a complex and unfamiliar situation with no clear limit and boundary, which made it difficult to provide health services. They expressed this complexity using various terms, including the terrible monster, the real battlefield, the devastating flood, and the disturbing tsunami in various socio-political and economic dimensions. From their point of view, this complex situation had caused confusion and panic among the medical community and lay people. According to the participants, this emerging crisis is the clash of cultures in society.

“*We were all confused in practice. The personnel were escaping the COVID-19 patients. Radiology personnel were not afraid to take a portable photo of a patient suspected with COVID-19. 20 % of the careless people seemed to negate the efforts of the other 80 % who follow the health protocol.“(Participant 2)*.

### 2. Individual and managerial competencies

The second main theme in the present study was managerial competencies. Having individual and managerial competencies played an important role in managing the crisis. This main category includes two subcategories of individual and managerial competencies.

#### 2.1 Individual competencies

Participants believed that dealing with the crisis required senior managers with competencies beyond general managers. Most participants emphasized such competencies as updatedness, far-sightedness, perseverance and seriousness, selflessness, experience and skill, knowledge and courage, sense of responsibility, compassionate work, and empathy with the patient, for an organization and the society to manage the crisis. Here are some of the statements of interviewees.

*“We have to be brave. We must not give up. We must have courage. “We fully took great care of the patients, the affected colleagues in the COVID-19, ward patients’ relatives and members of the society and provided consultation. Compassion should be the same for all patients. A 60-year-old patient is also the parent of a family” (Participant 9).*

#### 2.2 Managerial competencies

Based on the participants’ experiences, managing the COVID-19 crisis requires familiarity with the principles of teamwork. The participants referred to the need for collective wisdom to control the crisis. Participants believed that forming joint teams allowed them to share experiences to reach the goal rapidly with the least damage. They also referred to the need to anticipation and immediate action in all areas of the crisis management. According to the interviews, the managerial competencies required for the crisis management were expressed as follows:

*“Everything in society is like a football field. On the football field, everyone’s duty and role in the team is clear. While managing the COVID-19 crisis, everything must be in place. The scientific committee must stop the physician who prescribes CT scans frequently.“ (Participant 7)*.

Another participant stated the importance of teamwork in managing the COVID-19 crisis:

*“If you work individually, it is like a person who closes his/her eyes and crosses the street. Even if they arrive safe, their work is wrong and there may be a problem in the future. There is a need for teamwork. We have been in contact with other universities to understand their problems, and thus we can anticipate similar problems, use the right solutions, and have more peace of mind. Crisis management is required in all areas, including equipment, manpower, and physical space…predicting…predicting…predicting.“ (Participant 11).*

### 3. Comprehensive, accountable, and efficient management

The third main category was comprehensive, accountable, and efficient management in the face of the COVID-19 crisis, which included two subcategories: comprehensive and accountable management and efficient management.

#### 3.1 Comprehensive and accountable management

Comprehensive and accountable management was one of the characteristics of these participants. Summary of the participants’ opinions in this sub-category included intersectoral coordination, timely awareness with insight, use of the capabilities of key people in the society, full-time accountability, enhancement of public participation, use of all existing capacities in society, movement towards construction and production in supplying equipment, integration of experienced and young people, rapid use of all experiences, integration and coordination of health system, mobilization of faculty members and field activities, and quick assignment of the tasks of organizations and departments. According to the interviews, comprehensive and accountable management for tackling the crisis was stated as follows:

“*Awareness-raising should be timely and insightful and create the required concerns, not traumatizing and as a result paralyzing the society. We tried to bring all the key people with us, including the governor, prosecutor, religious leaders of the society, mayor, and so on. We have set up a 24-hour response system that is still going on. There is a weekly response program with the health staff and faculty members. Faculty members of the department of basic sciences were involved in the preparing disinfection solutions, field activities and community awareness and visiting various municipal service centers” (Participant 19).*

Another participant stated:

*“The chancellor of the university provided direct online responses every day to warn the community to behave based on the protocols. In addition to presenting a report on the situation in the city, he invited people to follow the protocols every day via voice messages. We tried to contact other ministries… Ministry of Industry, Mine and Trade, and Ministry of Defense to address the shortages and required equipment. We have created a management balance among all the components of the health system to reflect the result of this coordination in the management of all matters” (Participant 4).*

#### 3.2 Efficient management

Efficient management was another characteristic of these participants. Their opinions regarding this sub-category are categorized into hospital management, equipment management, manpower management, timely management with knowledge and updatedness, jihadist and self-sacrificing management, intersectoral communication management, fair management, neighborhood-based management, and creative management. Here, we present some key statements regarding efficient management:

*“We tried to use every corner of the hospital space and isolate the COVID-19 patients. We changed the use of the gym to an outpatient service center (16 hours). To provide equipment, we prepared equipment such as masks and shields with the help of the public. We had a shortage of manpower. We solved the problem by amending the recruitment rules. We also used jihadi volunteer forces” (Participant 13).*

On a similar note, another participant stated that:

*“My son contracted the COVID-19. I was following up on the phone and I could not leave the city and the people,“ said one university dean. With the help of jihadi forces, we tried to identify the contaminated areas from neighborhood to neighborhood, so that we left no blind spots. We accelerated the construction of the required equipment and moved towards sustainable construction. We tried to distribute all diagnostic and care services fairly” (Participant 6).*

### 4. Professional and organizational self-efficacy

The fourth and final main category included professional organization and self-efficacy in the management of the COVID-19 crisis, which included two sub-categories of professional self-efficacy and organizational self-efficacy.

#### 4.1 Professional self-efficacy

Based on the participants’ experience, being at the heart of the COVID-19 crisis management has improved their professional self-efficacy. Comprehensive and efficient management and control of the disease and reduction of mortality of the disease have made them feel satisfied. Self-reliance and increase in public participation also played a major role in the participants’ sense of empowerment and self-efficacy.

*“I gained as much as twenty years of experience during these twenty months. The feedback we get from society and the public satisfaction gives us strength. No one can replace the close experience we gained during the COVID-19 management. We touched everything in a real sense. My skills have developed.“ (Participant 17).*

On a similar note, another participant stated that:

*“The COVID-19 was a different experience for us. In other crises, such as flood and earthquake, we were in danger, but in the case of the COVID-19, the danger surrounded all health personnel, and no one was safe.“ (Participant 20).*

#### 4.2 Organizational self-efficacy

Based on the participants’ experience, being at the heart of the COVID-19 crisis management has led to self-efficacy in the organization. Summary of the participants’ views on organizational self-efficacy included reducing the need and dependency, coordinating all medical group specialties, forming scientific groups, developing intersectoral communication, and strengthening management interfaces.

*“The COVID-19 made all medical groups visible in crisis management. Before, there was always a focus on orthopedics and surgery. In the COVID-19 crisis management, other medical groups such as infectious disease specialists, anesthetists and others were also seen; they all work together to eliminate the challenges” (Participant 14).*

On a similar note, another participant stated that:

*“We formed a scientific group for sharing our experiences and ideas. Online university conferences in different management levels are a very useful experience for the crisis management. Managerial meditators need to be strengthened. First-level managers need to gain the trust of interface managers because people are in touch with interfaces, and they can have more influence on people’s compliance with protocols.“ (Participant16).*

## Discussion

This study was conducted to explore the perception of senior managers during COVID-19 Crisis in Iran using a qualitative method. According to the research results, there was various structural, cultural, and educational challenges in the face of COVID-19 crisis for the participants.

The health system in Iran is entangled with an unprecedented crisis of the Corona virus; the crisis caused by the infectious pandemic has opened up very different dimensions of management to the country’s health system. Senior managers in the health system are at the forefront of dealing with this disease and have a lot of experience in managing this crisis. This study explored the experience of senior managers in managing this crisis. The results of the present study showed that senior executives in the Iranian health system encountered several issues and challenges in the COVID-19 crisis, which could lead to the spread of disease and increased mortality. When there are structural challenges in the face of the crisis and it is not possible to provide manpower, medical equipment, medicine and a suitable space for hospitalization and treatment of the patient, the health of the community is endangered. Most participants in this study reported surprise and unpreparedness to provide medical equipment for the pandemic. These conditions can be seen in the health sector of some other countries, including India [15]. In Iran, sanction and economic problems such as inflation can be important factors in limiting the budget allocation to the health sector to supply the medical equipment [7].

From the view of the participants, the structure of human resources in medical centers in Iran does not meet the crisis conditions caused by COVID-19. In addition, affliction with COVID-19, extreme fatigue, stress, and anxiety in health workers have exacerbated the problem of manpower. Studies by Poortaghi et al. (2021) and Buheji et al. (2020) also reported organizational factors including manpower and physical resource as important challenges in COVID-19 crisis management [16, 17].

One of the important dimensions affecting health is the cultural dimension. Cultural issues are one of the major challenges is the expansion of the Corona in Iran. In the study of Buheji et al. (2020), cultural factors including lack of unity in procedures and cohesion between departments in crisis management as well as normalization and negligence of people in observing health protocols and standards are reported as effective challenges on crisis management in COVID-19; this is consistent with the results of the present study [18]. The results of the study by Priyadarsini et al. (2020) also showed that non-compliance with health protocols by people in the community has been reported as one of the most important cultural factors affecting the management of the COVID-19 crisis study [19]. In terms of cultural challenges, several factors can be effective as cultural problems in the development of COVID-19 in Iran, which can be attributed to issues such as distrust in health managers [20] and low perception of the risk [21] pointed out.

These cultural problems have caused people not to distrust managers even in a crisis like COVID-19, ignore the protocols, and attend family gatherings. Incredulity and low perception of the risk can be seen even among the authorities. Many people and officials in Iran do not believe in the disease until they get infected and experience it. This can be seen in the lack of quarantine in Qom province, as the first province in Iran which faced the COVID-19 problem. This can also be seen in the threefold increase in the number of hospitalized patients due to COVID-19 delta strain in the fifth wave of the disease in August 2021 compared to previous waves in Iran. On the other hand, some attitudes and tendencies to attend religious ceremonies that require the presence of a group such as congregational prayers and mourning ceremonies of religious leaders also make people feel vulnerable or accept attending ceremonies even at the cost of illness and death [22].

Therefore, despite the enactment of laws by the legislature to avoid participating in cultural gatherings and rituals and holding mourning ceremonies and other gatherings, we still see a large presence of people in these cultural programs. Omidi et al. (2020) also mentioned the lack of belief in vulnerability to coronavirus as one of the reasons for the spread of coronavirus in Iran. [23].

In crisis management, education is of fundamental importance. One of the challenges for senior executives during the COVID-19 crisis was the training challenges. Most participants stated that teamwork training in academic settings in Iran is poor. They believe that courses on continuing education for crisis management are not presented satisfactorily and have not prepared them for such crises. From the perspective of these participants, the logistical weakness of knowledge is observed during the COVID-19 crisis; also, despite numerous studies, important questions have not been answered so far. These participants believe that they have experienced organizational routine and, ultimately, surprise and poor performance instead of up-to-date research-oriented programs. The results of the study of Mohammadi et al. (2021) also showed that the lack of effective and continuous training programs in crisis management, especially biological crises such as COVID-19 pandemic, lack of preparedness maneuvers in the field of viral infectious diseases and weakness in teamwork between organizations involved in crisis management are the most important structural and educational challenges in COVID-19 crisis management; this finding is consistent with the results of the present study [24]. Due to the numerous structural, cultural, and educational challenges in Corona pandemic, the participants considered Corona as an arena of cultural confrontation, and the higher the culture of the society, the more successful it will be in dealing with Corona and the better its behavior in preventing and controlling the disease.

According to the findings, dealing with the COVID-19 crisis requires managers with individual and professional competencies beyond general managers. In this study, most participants agreed on individual competencies such as being up-to-date, far-sighted, perseverant and serious, and having self-sacrifice, experience and skill, knowledge and courage, sense of responsibility and compassion and empathy with the patient. As viewed by them, the use of collective wisdom, optimal use of time, quick prediction and action, and rapid decision-making combined with calmness are also influential in Covid crisis management. In this regard, the results of the study of Pedrosa et al. (2020) and Tsay et al. (2020) also showed that the experience and knowledge of personnel, up-to-date scientific knowledge, decision-making skills and effective performance in critical and complex situations, time management and use of the colleagues’ experience and knowledge have important roles in managing complex and unpredictable crises [25, 26]. With further reflection, we realize that these are two categories of competencies that can be developed through education. Therefore, it is suggested that senior managers consider these competencies should make an attempt to develop and improve their competence, so that they can react more appropriately in the face of the Corona crisis.

This study showed that COVID-19 crisis management required comprehensive and accountable management. Most participants believed that coordination between different sectors should be set to manage the COVID-19 crisis. In Iran, many measures have been taken to move the country’s crisis management system step by step towards becoming more coordinated with the approach of creating a coordinated and integrated crisis management system; In this regard, the “Law on the Establishment of the Crisis Management Organization” has been developed with the focus on coordinating the relevant organizations.

Despite the fact that this law correctly emphasizes the issue of coordination and pays attention to all levels, still the main weakness of the country’s crisis management is its inter-organizational and even intra-organizational coordination. Among the lack of coordination within the organization is the overcrowding of patients in pharmacies to access drugs related to COVID-19. Despite the need for coordination and coherence between executive agencies during the Corona crisis, there is a frequent lack of coherence and different approaches in various organizations. For example, in the fifth wave of the Corona, the Ministry of Health offered a two-week quarantine, but the Corona Crisis Headquarters did not approve it, or when a metro closure was offered to prevent an outbreak in Tehran, it was not accepted.

Participants in the study emphasized the need to mobilize people to manage the Corona crisis. They also mentioned educating the public in this regard as the most important way to fight the disease. According to the participants, key people in the society, media, education, non-governmental organizations, and relevant executive bodies have an important role in educating people. Dealing with the crisis requires public determination, without which we cannot get rid of the corona virus transmission chain. Therefore, public responsibility towards Corona is a necessary fact which is achieved through awareness [25]. Based on their experiences, the participants stated that it was necessary to employ managers who have both experience and youthful strength in managing Corona crisis. Findings of the study of Mohammadi, et al. (2021) showed that one of the priorities of medical center managers who work in critical situations is the experience and concern of the healthcare personnel who deal with COVID-19 patients [24].

Another result of this study was coordination between the health and treatment sector. Despite the fact that general health policies have emphasized the issue of the priority of prevention over treatment in Iran in the last two decades, the dominant approach of the health system was adopting a treatment-oriented approach; health activities were carried out separately, with no connection with the treatment system. However, optimal health systems are integrated, where prevention and treatment information is available in a coordinated manner. Health island practices declined with the Corona pandemic. Corona integrated many health activities (such as diagnosis) to the treatment system and made it possible to follow up the disease identified by the health system through screening.

Another result of this study was conscious management in the face of the Corona crisis. One of the approaches of conscious management in Iran has been the application of the neighborhood-based management approach in the Corona crisis. The main goal of this approach was disease management, and its specific objectives included diagnosing and screening the patients, reducing the incidence of the disease by cutting the transmission chain, and reducing the hospitalization rate and mortality of patients with COVID-19. This project was carried out through active participation of the people, Militia, Red Crescent, pre-hospital emergency, and executive and non-governmental organizations in the country. In this plan, screening and identifying the cases were done to break the chain of disease transmission. In this project, three groups worked in three areas: 1) support teams focusing on Militia and the Red Crescent, with the aim of supporting the elderly and incurable patients and providing them with medicine and livelihoods, and attracting and directing public aid toward occupational support; care teams to track the patients; and environmental health-focused monitoring teams to intensify the inspections and visits. The community-based model is a bottom-up management approach that focuses on people’s participation in resolving crisis-related situations.

In fact, its purpose is to reduce the vulnerability of communities and strengthen the ability and participation of people to deal with the risks of the crisis [27, 28]. The results of the study of Mohammadpour et al. (2021) also showed that inter-sectorial and organizational coordination, inter-sectorial cooperation, use of all potentials in society, involvement of the general public, and optimal management of equipment and human resources were important factors which influence effective crisis management during the COVID-19 crisis [29]. The results of Sharifia et al.‘s (2020) study also showed that comprehensive and efficient management was the key to success in the COVID-19 crisis. Although many factors can be effective in crisis management, efficient and effective management is one of the most important key factors in tackling complex situations, including COVID-19, which overshadows other factors [30].

The majority of the participants in the study cited jihadi management as a conscious management approach to the Corona crisis. Jihadi management included using the capacity of the public to deal with the Corona, which led to the activation of religious and public organizations, mosques, charities, and some private companies with widespread distribution throughout the country and a focus on low-income areas; it provided access to health items and some medical services. In jihadi management, people value serving others. This type of thought arises from religious beliefs [31].

Self-efficacy was another finding of the present study in the management of COVID-19 crisis. Based on the experience of the participants, the COVID-19 crisis has led to their self-efficacy. Comprehensive and informed management in order to provide prevention and treatment services has resulted in their satisfaction. Self-reliance and engaging people with oneself also played a major role in participants’ sense of empowerment and self-efficacy. According to Bandura theory, one of the most important sources of self-efficacy is successful actions (Bandura). Gaining successful experiences in managing challenging situations can lead to promotion of the managers’ self-efficacy [32]. The results of Jafarpourian et al.‘s (2016) study also showed that managers with a history of presence in critical situations had significantly higher self-efficacy than other managers [33].

Based on the experience of the participants, the COVID-19 Crisis has led to organizational self-efficacy, including reduced need and dependency, coordination of all medical specialties, formation of scientific groups, development of cross-sectorial communication, and reinforcement of managerial interfaces. The results of the study by Thakur et al. (2020) and Rivaz et al. (2020) also showed that although the Covid-19 crisis made challenges and threats to the managers and employees of the health system, this crisis brings valuable opportunities and achievements such as empathy, cohesive teamwork between physicians and healthcare team, effective inter-sectorial communication, collaboration between managers and staff, optimal use of facilities and equipment, and trust in professional and organizational capabilities [34, 35]. Thus, in this challenging path of Corona crisis, organizations become self-sufficient by overcoming the challenges by competent and capable managers.

## Limitations

This research also had its limitations. Despite the necessary coordination and determination of the time and place of the interview, there was often a need to interrupt the interview process and reschedule the interview due to the managerial role of the participants in the management of Corona. In addition, there was no intimate atmosphere between the researcher and participants due to the need to follow the protocols and social distance. This study is the result of interviews conducted in the crisis response phase and may provide other new experiences for senior managers after the crisis and in the reconstruction phase. In this study, only individual interviews were used to collect the data, and it was not possible to use other methods such as focused interviews due to the Corona crisis.

## Study strengths

The present research is the first study with a qualitative approach carried out to investigate the experiences of crisis management from the perspective of senior university administrators in the COVID-19 crisis in Iran, which can provide useful information for policymakers to properly plan crisis management in emerging diseases. Variety in sampling and selecting different managerial jobs was another strength of this study.

## Conclusions

The results of the study show that structural, educational, and cultural challenges are involved in the management of COVID-19 crisis. Therefore, it is necessary to take the required interventions to address the challenges and provide a suitable cultural and organizational context to prevent the spread of this disease. This study showed that the personal and managerial competencies are two types of capabilities a senior manager needs in dealing with crises. According to the results of the present study, jihadi management using public forces and neighborhood-based management in crises were the strengths in the management of Corona crisis. Therefore, health system managers and policy- makers can use the above-mentioned approaches to better plan and effectively manage the COVID-19 pandemic.

## Data Availability

The datasets used and /or analyzed during the current study are available from the corresponding author on reasonable request.
